# Breaching the immune-cold barrier in pMMR/MSS metastatic colorectal cancer: emerging strategies beyond standard care

**DOI:** 10.3389/fimmu.2026.1773002

**Published:** 2026-03-13

**Authors:** Wenkai Kang, Zhiqiang Zhang, Wentao Li, Qingyuan Zhang, Ningning Li, Youheng Jiang, Junjing Zhang, Yulong He

**Affiliations:** 1 Digestive Diseases Center, Guangdong Provincial Key Laboratory of Digestive Cancer Research, The Seventh Affiliated Hospital, Sun Yat-sen University, Shenzhen, China; 2 Tomas Lindahl Nobel Laureate Laboratory, The Seventh Affiliated Hospital, Sun Yat-sen University, Shenzhen, China; 3 Department of Burns and Plastic Surgery, The Seventh Affiliated Hospital, Sun Yat-sen University, Shenzhen, China; 4 Future Medical Center, Shenzhen University of Advanced Technology, Shenzhen, China; 5 Department of Hepato-Biliary Surgery, Department of Surgery, Hohhot First Hospital, Hohhot, China

**Keywords:** late-line immunotherapy, liver metastasis, metastatic colorectal cancer, tumor microenvironment, vascular normalization

## Abstract

Mismatch repair–proficient (pMMR) or microsatellite stable (MSS) metastatic colorectal cancer (mCRC) remains largely refractory to immunotherapy due to its intrinsically immunologically cold tumor microenvironment. Although frontline chemotherapy combined with immune-induction strategies can improve progression-free survival, these benefits have not translated into overall survival gains. Importantly, late-line trials such as LEAP-017 suggest that biologically meaningful immune activity may be masked by dilution effects in unselected populations. This review advocates a shift from empirical treatment approaches toward mechanism-driven precision stratification in refractory pMMR/MSS mCRC. We highlight two complementary immune activation strategies: tumor microenvironment remodeling via tyrosine kinase inhibitor-mediated vascular normalization, and intensified immune priming through cytotoxic T-lymphocyte-associated protein 4 blockade. To address persistently low response rates, we propose an integrated precision framework incorporating genomic repurposing, anatomical filtering, and multimodal synergy, aiming to translate transient immune activation into durable survival benefit.

## 1 Introduction

### 1.1 The clinical landscape: the cold microenvironment

The current therapeutic landscape of metastatic colorectal cancer (mCRC) is characterized by a significant polarization in survival outcomes. On one hand, the advent of immune checkpoint inhibitors (ICIs) has revolutionized the treatment paradigm for patients with mismatch repair-deficient (dMMR) or microsatellite instability-high (MSI-H) tumors, conferring durable survival benefits to this subgroup, which constitutes approximately 5% of cases (1, 2).However, for the vast majority (approximately 95%) of patients harboring mismatch repair-proficient (pMMR) or microsatellite stable (MSS) tumors, clinical benefits remain extremely limited (3, 4).This resistance is fundamentally driven by an intrinsically immunologically cold microenvironment, phenotypically defined by a paucity of tumor-infiltrating lymphocytes and a highly suppressive myeloid stroma.

The clinical consequences of this resistance are particularly severe. For patients who have progressed after standard cytotoxic chemotherapy (fluorouracil, oxaliplatin, irinotecan) and targeted agents (anti-vascular endothelial growth factor (anti-VEGF) or anti-epidermal growth factor receptor (anti-EGFR) monoclonal antibodies), subsequent treatment options are scarce (5). Current standard of care (SOC) options for late-line treatment (specifically defined as third-line therapy or beyond, following progression on standard fluoropyrimidine, oxaliplatin, and irinotecan-based regimens), such as Regorafenib or Trifluridine/Tipiracil (TAS-102), offer only marginal survival advantages, with median overall survival (OS) typically limited to a plateau of 6 to 8 months (6, 7). This significant therapeutic ceiling highlights the most urgent unmet clinical need in contemporary gastrointestinal oncology: the development of novel strategies capable of overcoming this intrinsic primary immune resistance.

To overcome this barrier, we must first redefine its biological nature. Traditionally, pMMR/MSS tumors have been classified as immunologically cold simply due to their low tumor mutation burden (TMB) and paucity of effector T-cell infiltration (8–11). However, recent immunogenomic insights reveal that this is not a passive state, but an active, multi-layered exclusion sustained by specific molecular mechanisms. First, at the intracellular signaling level, aberrant pathways such as PI3K/Akt/mTOR actively repress immune recognition and orchestrate an immunosuppressive microenvironment (12). Second, host-intrinsic genetic factors play an underappreciated role; specific polymorphisms in microRNAs (e.g., *miR-27a*) have been shown to modulate systemic immune susceptibility, thereby influencing individual progression trajectories (13). Finally, at the structural level, emerging data highlight B7-H3 (CD276) as a pervasive gatekeeper on tumor vasculature that physically and functionally blocks T-cell penetration, independent of MSI status (14). Consequently, the resistance observed in the clinic is the cumulative result of these genomic, signaling, and structural barriers (15).

### 1.2 The efficacy paradox: the failure of standard upfront priming

To breach the immune barrier of pMMR/MSS tumors, early attempts primarily focused on the first-line treatment setting (16). The mechanistic rationale was theoretically sound: utilizing cytotoxic chemotherapy to induce immunogenic cell death (ICD) as a trigger, anti-angiogenic agents (e.g., bevacizumab) as a promoter, and superimposing ICIs as an effector, with the aim of synergistically initiating anti-tumor immunity.

However, this strategy encountered a significant efficacy paradox under rigorous clinical scrutiny. A recent meta-analysis published by Ando et al. (2025), which included four pivotal randomized controlled trials such as AtezoTRIBE and CheckMate 9X8, provided a crucial evidence-based foundation for evaluating this hypothesis (17).

Data revealed a clear clinical dilemma: although adding ICIs to standard first-line chemotherapy (with or without bevacizumab) did yield a statistically significant improvement in progression-free survival (PFS) (hazard ratio [HR] 0.82, 95% CI: 0.70–0.96, *P* = 0.01), indicating that this combination can indeed transiently suppress tumor progression, this failed to translate into an OS benefit (HR 0.91, 95% CI: 0.74–1.12, *P* = 0.39). Furthermore, this marginal PFS benefit was accompanied by a significantly increased toxicity cost, with a marked rise in the incidence of grade 3 or higher adverse events (17).

This disconnect between PFS benefit and OS stagnation reveals an important biological insight: the immunomodulatory effects generated by chemotherapy and anti-angiogenic agents appear to be of a transient nature. While sufficient to delay progression initially, they fail to induce the kind of durable, self-sustaining anti-tumor immunity capable of fundamentally altering the disease course. This strongly demonstrates that in pMMR/MSS tumors, simple upfront addition strategies face a clear therapeutic bottleneck, compelling us to shift our focus toward novel mechanistic breakthroughs in late-line settings (17). Clinically, the lack of OS benefit may be partially attributed to the impact of subsequent lines of therapy, which can dilute the statistical separation between arms (18). Biologically, however, it suggests that the immunomodulatory effects generated by chemotherapy and anti-angiogenic agents are likely transient rather than transformative (19). While sufficient to delay progression initially (improving PFS), this induction strategy may fail to eradicate the dormant, drug-tolerant persister cells responsible for long-term relapse. Furthermore, the selective pressure of cytotoxic agents could potentially accelerate clonal evolution, promoting the emergence of resistant subclones that are impervious to late-line immune control (20). Thus, simple upfront addition strategies face a mechanistic bottleneck: they treat the bulk tumor but fail to fundamentally reprogram the immunosuppressive soil required for durable survival.

### 1.3 A paradigm shift: from upfront priming to late-line activation

**Figure 1:**
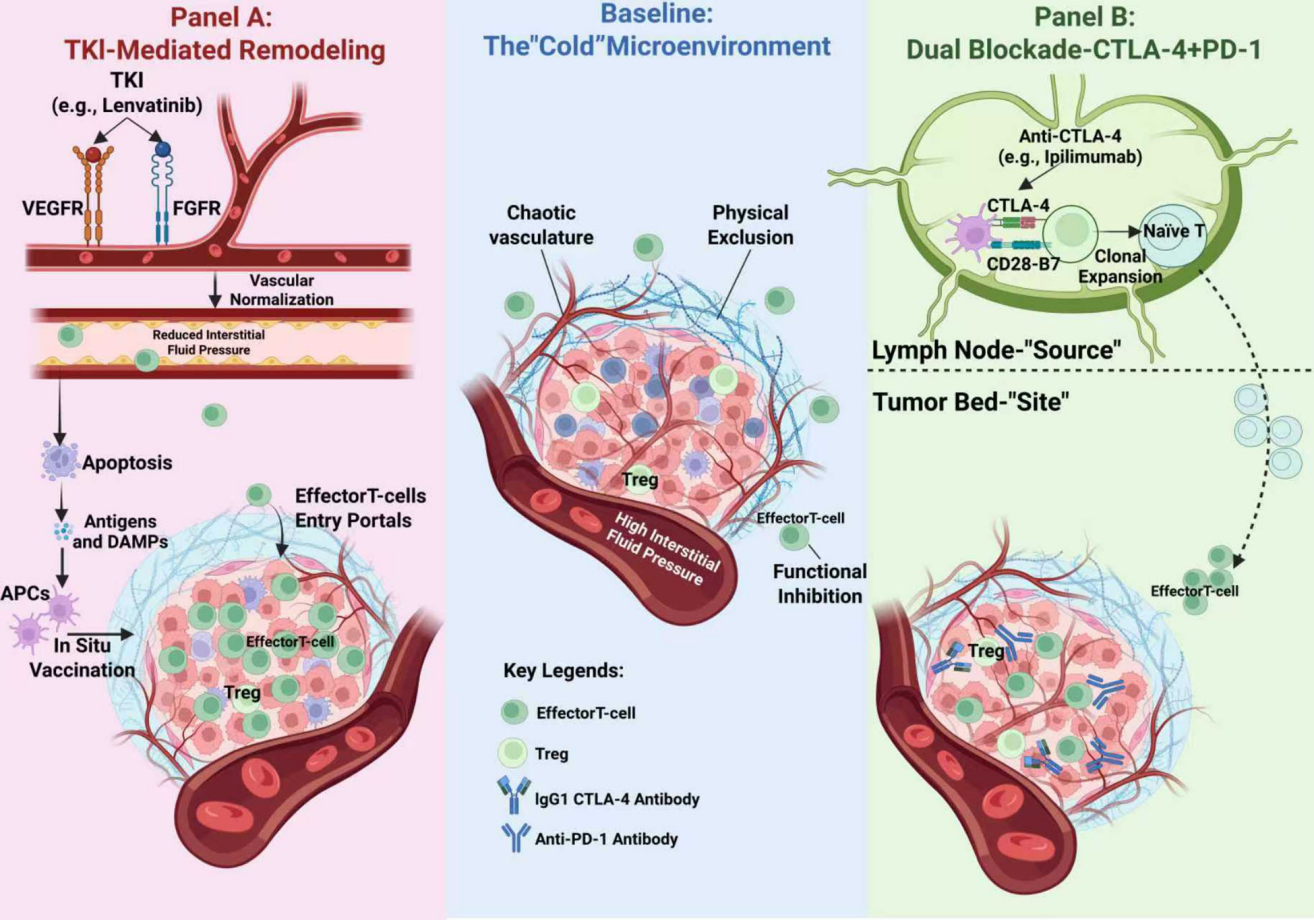
Distinct mechanistic pathways for breaching the immune-cold barrier in pMMR/MSS mCRC. The central panel illustrates the baseline cold microenvironment, characterized by chaotic vasculature causing physical immune exclusion and high interstitial fluid pressure, alongside functional inhibition by regulatory T cells (Tregs). **(A)** Strategy I: TKI-Mediated Remodeling. Multi-target TKIs (e.g., Lenvatinib) targeting VEGFR/FGFR pathways induce vascular normalization, creating a physical portal for T-cell entry. Concurrently, TKI-induced apoptosis acts as an *In Situ* Vaccine, releasing antigens and DAMPs to activate APCs. **(B)** Strategy II: Dual Blockade (CTLA-4 + PD-1). This strategy operates on two spatial levels: in the Lymph Node (Source), CTLA-4 blockade restores CD28-B7 co-stimulation to drive the clonal expansion of naïve T cells; in the Tumor Bed (Site), IgG1-based anti-CTLA-4 antibodies deplete immunosuppressive Tregs via ADCC, facilitating the influx of *de novo* effector T cells.

Given the limitations of upfront priming strategies, the exploration of immunotherapy in pMMR/MSS mCRC necessitates an urgent strategic realignment. When patients develop resistance to standard cytotoxic drugs and targeted therapies, the therapeutic goal shifts from merely delaying disease progression to exploiting novel mechanistic windows to overcome entrenched immunosuppression. Against this backdrop, this review focuses on the late-line refractory setting, exploring two emerging and mechanistically complementary immune activation pathways (**Figure 1**).

The first pathway focuses on Microenvironment Remodeling. This is primarily embodied by the combination of tyrosine kinase inhibitors (TKIs) and ICIs. In this strategy, TKIs serve not merely as anti-angiogenic agents but as microenvironment modulators (21, 22), aiming to physically breach the barrier of immune exclusion via vascular normalization and the reversal of immunosuppressive signals (23).

The second pathway focuses on Intensified Immune Priming. This is exemplified by the application of dual immune checkpoint inhibitors (Dual ICI, i.e., anti-programmed cell death protein 1/ligand 1 (anti-PD-1/L1) combined with anti-cytotoxic T-lymphocyte-associated protein 4 (anti-CTLA-4)). Unlike simple PD-1 blockade, the introduction of a CTLA-4 inhibitor aims to reactivate T cells during the priming phase in lymph nodes, thereby overcoming the T-cell exhaustion and priming failure common in monotherapy (24, 25).

### 1.4 Core hypothesis and objectives

Current clinical data present a clear dichotomy. While phase III trials such as LEAP-017 confirm that unselected immunotherapy fails to improve OS in the general pMMR/MSS population, durable responses persist in a reproducible minority (~10%). This dissociation—statistical negativity versus biological activity—forms the empirical basis of our analysis.

Conceptually, we hypothesize that immune coldness in pMMR/MSS mCRC is not a uniform phenotype but stems from distinct, often mutually exclusive barriers: either physical vascular exclusion or functional priming failure. The primary objective of this review is to translate this mechanistic heterogeneity into a clinical stratification framework. By integrating genomic markers (specifically repurposing *RAS* and identifying *POLE/POLD1* mutations) with anatomical filters (liver metastasis status), we aim to define precise therapeutic niches, moving clinical practice beyond the current all-comers impasse. The following sections will detail the mechanistic logic and clinical evidence for these targeted strategies.

## 2 Strategy I: TKI-mediated microenvironment remodeling

Unlike frontline chemotherapy, which primarily relies on cytotoxicity to directly kill tumors, the primary strategic goal in the refractory setting undergoes a fundamental shift: from triggering cell death to systemically reversing the immunosuppressive signals that maintain the cold microenvironment (26). The core of this strategy lies in utilizing multi-target TKIs as microenvironment modulators, rather than acting solely as anti-angiogenic agents. By targeting key signaling pathways such as VEGF and fibroblast growth factor receptor (FGFR), TKIs can disrupt the inherent defensive architecture of pMMR/MSS tumors, creating a physical window conducive to immune infiltration for subsequent or concurrent immune checkpoint blockade (27, 28).

### 2.1 Mechanistic rationale: vascular normalization and immune infiltration

Mechanistically, the immune resistance of pMMR/MSS mCRC is maintained by a dual barrier: first, physical exclusion, where a chaotic tumor vasculature impedes the infiltration of cytotoxic T lymphocytes into the tumor stroma (29, 30); and second, functional inhibition, where high concentrations of suppressive factors within the microenvironment limit T-cell activity (31). In this context, the mechanism of action of TKIs extends beyond the traditional paradigm of starvation therapy.

The cornerstone of this strategy lies in the Vascular Normalization hypothesis (32). By pruning immature, chaotic vessels and lowering interstitial fluid pressure, TKIs restore the originally leaky tumor vascular network into an organized, perfusion-competent structure (33). This process is critical as it transforms the vascular endothelium from a physical barrier into a “portal” permissive to immune cell trafficking, thereby significantly enhancing the transport efficiency of effector T cells into the tumor parenchyma (34). Fundamentally, this represents a pharmacological reversal of the “immune-excluded” phenotype, restoring the accessibility of an otherwise impermeable microenvironment to immune effector cells.

#### 2.1.1 Breaking the physical barrier: from starvation to vascular normalization

In traditional oncological cognition, the goal of anti-angiogenic therapy is often conceptualized as starvation therapy, which aims to inhibit tumor growth by severing its blood supply (35). However, in the context of combined immunotherapy, the pursuit of vascular blockade alone may prove counterproductive, as therapy-induced excessive hypoxia can exacerbate immunosuppression within the microenvironment (36, 37). Therefore, the key mechanism of TKIs in this setting is not simple vascular blockade, but rather the promotion of a more orderly Vascular Normalization (38).

pMMR/MSS tumors typically exhibit a chaotic, leaky, and immature vascular network, resulting in abnormally elevated interstitial fluid pressure (39). This high-pressure state physically impedes the infiltration of drugs and immune cells from the vasculature into the tumor parenchyma, thereby maintaining the so-called immune exclusion phenotype (40, 41).

By inhibiting the VEGF/vascular endothelial growth factor receptor (VEGFR) pathway, small-molecule TKIs (e.g., Lenvatinib) can prune these immature vessels and restore the structural integrity of the vasculature (42). This process is pivotal as it transforms the vascular endothelium from a dense physical barrier into a portal permissive to immune cell passage. This structural repair significantly enhances the trafficking efficiency of effector T cells, enabling them to cross the vessel wall and deeply infiltrate the tumor parenchyma, thereby providing the necessary effector reservoir for subsequent immune checkpoint blockade (43).

#### 2.1.2 Reversing immunosuppression: *in situ* vaccination and microenvironment reprogramming

Having overcome the physical barrier, the second challenge lies in addressing the intrinsic antigen invisibility and functional inhibition of pMMR/MSS tumors (44). Since these tumors typically possess a low TMB and lack sufficient neoantigens to trigger immune recognition, they remain in a state of immunological silence or invisibility under immune surveillance (45).

Here, TKI-mediated microenvironment remodeling functions in a manner analogous to an *in situ* vaccination. Specific TKIs, such as lenvatinib and regorafenib, are capable of inducing ICD, a process accompanied by the release of damage-associated molecular patterns (DAMPs) and previously sequestered tumor antigens (46).These molecules serve as critical danger signals, recruiting and activating antigen-presenting cells (APCs) to induce a *de novo* antigen-specific T-cell response (47).

Furthermore, sustained activation of the VEGF pathway is known to inhibit the maturation of dendritic cells (DCs) and promote the recruitment of regulatory T cells (Tregs) (48). By blocking this signaling axis, TKIs not only increase antigen release but also systemically alleviate these suppressive mechanisms, functionally resetting the immunogenicity of the tumor microenvironment (49). This dual physical and biochemical remodeling constitutes the fundamental mechanistic basis for the TKI plus ICI strategy to overcome primary resistance.

#### 2.1.3 Optimization: pharmacological determinants of normalization

To effectively translate vascular normalization into clinical benefit, treatment protocols must pivot from maximum toxicity to optimal biological modulation. Regarding drug selection, multi-kinase inhibitors (e.g., Lenvatinib or Regorafenib) appear superior to highly selective VEGF antibodies (e.g., Bevacizumab) in this refractory setting. This advantage stems from their broader inhibitory profiles; by simultaneously targeting FGFR and colony stimulating factor 1 receptor (CSF1R), these agents counteract the redundant angiogenic pathways and macrophage recruitment that typically drive resistance to pure VEGF blockade (28, 50).

Dosing and timing are equally critical. The Maximum Tolerated Dose standard in oncology may be counterproductive here, as excessive vascular pruning induces hypoxia and exacerbates immunosuppression (32, 33). Conversely, lower normalizing doses improve vessel perfusion and oxygenation, thereby facilitating T-cell infiltration (33). While preclinical models suggest that a sequential run-in period (administering TKI days before ICI) can prime the tumor bed, clinical implementation favors continuous concurrent administration to prevent the rapid rebound of abnormal vasculature that occurs once TKI pressure is lifted (32, 43).

### 2.2 Clinical evidence: the lesson from LEAP-017

In evaluating the clinical translational potential of TKI-mediated microenvironment remodeling strategies, the Phase III LEAP-017 trial offers a highly instructive case study. This study evaluated the efficacy of Lenvatinib plus Pembrolizumab versus standard of care (Regorafenib or TAS-102) in patients with refractory mCRC (50). Although the trial failed to meet its primary statistical endpoints, in-depth analysis of its data reveals the core dilemmas and potential value of this strategy.

#### 2.2.1 Biological signals amidst statistical negativity

From the perspective of the intention-to-treat (ITT) population, LEAP-017 failed to meet its primary endpoint of OS (median OS: 9.8 months *vs*. 9.3 months; HR 0.83; 95% CI: 0.68–1.02; *P* = 0.038, failing to cross the prespecified efficacy boundary). However, results from secondary endpoints revealed a significant phenomenon of dissociation between biological activity and OS benefit (50).

The combination arm observed a clinically meaningful objective response rate (ORR) of 10.4%, compared to only 1.7% in the standard of care arm. In the refractory pMMR/MSS population, significant tumor shrinkage in approximately 10% of patients serves as a crucial signal, suggesting that TKIs may have successfully unlocked the immune microenvironment in a subset of patients (50). Furthermore, the significant improvement in PFS (HR 0.69, 95% CI: 0.56-0.85) further confirms the activity of this combination in controlling disease progression.

#### 2.2.2 The dilution effect and the end of all-comers strategies

The phenomenon where ORR/PFS benefits failed to translate into OS benefits does not necessarily negate the mechanistic validity of TKI+ICI, but rather typically exemplifies the Dilution Effect. Specifically, the efficacy signal in a specific responsive subpopulation (approximately 10-15%) was statistically masked by the non-responders who constituted the vast majority of the ITT population (50).

LEAP-017 represents not merely a trial that missed its endpoints, but an important proof-of-concept: it confirms that reversing immune tolerance in pMMR/MSS tumors via pharmacological means is biologically feasible. However, it simultaneously highlights the limitations of the All-comers unselected model. The true lesson of this trial is that future success depends not on the drug itself, but on the ability to precisely identify that specific 10% subpopulation that benefits from microenvironment remodeling—a core issue addressed in Section 4 on precision stratification. **Table 1** provides a comprehensive summary of the pivotal efficacy and safety outcomes from the LEAP-017 trial. These data highlight a critical dissociation between the statistically significant improvement in PFS (HR 0.69, 95% CI: 0.56-0.85, *P* < 0.001) and the lack of OS benefit (HR 0.83, 95% CI: 0.68–1.02, *P* = 0.038). This discrepancy serves as key evidence illustrating how the biological activity of the TKI plus ICI combination—manifested by a clinically meaningful ORR—was likely diluted within the unselected intention-to-treat population, underscoring the necessity for the precise patient selection strategies proposed in this review.

**Table 1:** Summary of key efficacy and safety outcomes from the phase III LEAP-017 trial in refractory pMMR/MSS mCRC (50).

Endpoints	Lenvatinib + Pembrolizumab (N = 223)	Standard of care (N = 211)	HR (95% CI)/difference	*P*-value	Clinical interpretation
Median OS	9.8 months	9.3 months	HR 0.83 (95% CI: 0.68–1.02)	0.038	Did not cross the prespecified efficacy boundary; OS benefit was not statistically significant.
Median PFS	3.8 months	3.2 months	HR 0.69 (95% CI: 0.56–0.85)	<0.001	Significantly delayed disease progression, demonstrating biological activity of the combination.
ORR	10.4%	1.7%	+8.7% absolute difference	--	Approximately 1/10 of refractory patients achieved tumor shrinkage, suggesting sensitivity in a specific subpopulation.
Safety (≥Grade 3 TRAE)	Significantly increased	Baseline level	--	--	Higher toxicity associated with combination therapy; careful risk-benefit assessment is required.

CI, confidence interval; HR, hazard ratio; OS, overall survival; PFS, progression-free survival; ORR, objective response rate; SOC, standard of care (Regorafenib or Trifluridine/Tipiracil); TRAE, treatment-related adverse events. Note: Although the *P*-value for OS was 0.038, it did not meet the prespecified statistical significance boundary (*P* < 0.013) required for positive determination due to multiple testing adjustments. Data adapted from Kawazoe et al. (2024) (50).

## 3 Strategy II: intensified immune priming via dual blockade

While TKI-mediated strategies focus on overcoming physical exclusion, resistance mechanisms in some patients stem primarily from intrinsic defects in T-cell function—specifically, insufficient priming of naïve T cells or exhaustion of effector T cells (51). Addressing this challenge, the second emerging strategy shifts reliance away from vascular modulation toward a more intensified mode of immune intervention: Dual Checkpoint Blockade (52). The core of this strategy lies in introducing anti-CTLA-4 antibodies (e.g., Ipilimumab) to synergize with anti-PD-1/L1 therapy (46, 53). Its mechanistic basis rests on the premise that the insensitivity of pMMR/MSS tumors to PD-1 monotherapy is attributable not only to inhibition during the effector phase but, more critically, to a functional arrest in the early stages of the immunity cycle—namely, the antigen presentation and T-cell priming phases (46). Therefore, intensified priming via dual blockade aims to systemically reactivate this stalled cycle.

### 3.1 Mechanistic rationale: CTLA-4 and the reactivation of the priming phase

To understand the unique value of dual blockade in refractory pMMR/MSS mCRC, it is essential to mechanistically distinguish between the two key checkpoint targets. The PD-1/PD-L1 interaction occurs primarily during the effector phase in peripheral tissues, and its blockade aims to restore the effector function of infiltrating T cells (54, 55). However, cold tumors often lack pre-existing effector T cells, rendering PD-1 monotherapy ineffective due to a lack of substrate (56, 57).

In contrast, CTLA-4 operates primarily during the central priming phase within secondary lymphoid organs (58). During the early stages of T-cell activation, CTLA-4 competitively binds to B7 molecules on APCs, thereby inhibiting T-cell expansion (59).Introducing a CTLA-4 inhibitor into the treatment regimen essentially shifts the window of immune intervention upstream. It does not rely on pre-existing T cells within the tumor but is instead dedicated to promoting the clonal expansion and activation of naïve T cells and recruiting these *de novo* effector cells to the tumor site (60). This reactivation at the source constitutes the key mechanism by which dual immunotherapy overcomes primary or acquired resistance to PD-1 blockade.

#### 3.1.1 Targeting the lymph node phase: restarting the cycle at its source

The efficacy of PD-1 inhibitors is limited by their high dependence on pre-existing T-cell infiltration within the tumor microenvironment (61). In cold tumors lacking effector T-cell infiltration, blocking the PD-1 pathway alone is insufficient to effectively initiate an anti-tumor immune response (62, 63). The introduction of CTLA-4 inhibitors (e.g., Ipilimumab) aims to overcome this scarcity of effector T cells at the source.

CTLA-4 blockade occurs primarily in secondary lymphoid organs, rather than locally within the tumor (64). In normal immune physiology, CTLA-4 competitively binds to B7 molecules (CD80/86) on the surface of APCs, thereby blocking CD28-mediated co-stimulatory signals and curbing T-cell activation (45, 65).

By using anti-CTLA-4 antibodies, we not only block this inhibitory signal but, more importantly, restore the critical co-stimulatory signaling mediated by CD28-B7 (66, 67). This mechanism effectively promotes the clonal expansion and activation of naïve T cells within secondary lymphoid organs. This explains why dual blockade elicits responses in pMMR/MSS tumors: it does not rely on reversing the function of exhausted T cells within the tumor, but rather traffics *de novo*, highly proliferative specific T-cell clones into the tumor microenvironment.

#### 3.1.2 Overcoming acquired resistance: depleting Tregs

Beyond promoting the generation of new T cells, another key mechanistic advantage of introducing CTLA-4 inhibitors lies in the depletion of immunosuppressive Tregs (68, 69). Preclinical and clinical evidence indicates that during long-term PD-1 blockade, the tumor microenvironment often develops adaptive resistance, a prominent feature of which is the compensatory upregulation of Tregs. These Tregs construct a potent immunosuppressive barrier by secreting TGF-β and IL-10, leading to secondary resistance (70).

CTLA-4 is highly expressed on the surface of tumor-infiltrating Tregs (71). Since antibodies of the IgG1 isotype, such as Ipilimumab, possess Fc region effector functions, they can recruit macrophages or natural killer (NK) cells to directly kill and deplete these CTLA-4-high Tregs via antibody-dependent cell-mediated cytotoxicity (ADCC) (72, 73).

This clearance effect not only weakens the immunosuppressive barrier in the microenvironment but also restores the activity of effector T cells suppressed by Tregs. Therefore, in the refractory setting where PD-1 monotherapy has failed, dual blockade exerts a synergistic effect: clearing inhibitory Tregs locally (tumor microenvironment) while inducing a new round of T-cell expansion systematically (lymph nodes). This mechanistic complementarity constitutes the biological basis for the dual immunotherapy strategy to rescue patients with advanced pMMR/MSS tumors.

### 3.2 Clinical evidence in refractory settings: survival breakthroughs in the real world

Although dual checkpoint inhibitors (PD-1/L1 inhibitor combined with CTLA-4 inhibitor) face challenges in the unselected general population of pMMR/MSS mCRC, this strategy demonstrates significant survival benefits in the specific context of refractory salvage treatment.

Recent real-world evidence provides robust clinical support for immune intensification following acquired resistance. A pivotal study by Zhao et al. (2024) evaluated the efficacy of Dual ICI in heavily pretreated pMMR/MSS mCRC patients who had previously progressed on PD-1 inhibitor therapy. The results showed that the cohort receiving dual blockade salvage therapy achieved clinically meaningful survival benefits, with a median OS reaching 15.8 months (74).

To interpret this data within a clinical context, comparison with historical benchmarks is necessary. Currently, the historical median OS for third-line standard of care (e.g., Regorafenib or TAS-102) typically plateaus at 6 to 8 months (75). In comparison, the dual immune strategy nearly doubled the survival duration and was significantly superior to the concurrent control group (10.2 months; HR 0.59, 95% CI: 0.38-0.89; *P* = 0.017).

This significant survival advantage extends beyond mere statistical significance; it supports a key biological hypothesis: the biological characteristics of acquired immune resistance may differ fundamentally from those of primary resistance. For patients who have previously shown transient response or exposure to immunotherapy, intensified priming via the introduction of a CTLA-4 inhibitor holds the potential to effectively bridge the therapeutic gap following PD-1 monotherapy failure, offering a novel therapeutic window for this refractory patient population.

### 3.3 Critical appraisal: the narrow therapeutic index and patient selection

While dual blockade offers a potential salvage pathway, a critical examination of historical and emerging data reveals a narrow therapeutic index. The efficacy of this strategy must be weighed against its substantial toxicity burden, a lesson underscored by prior randomized trials. For instance, the CCTG CO.26 trial (76) (Durvalumab plus Tremelimumab) demonstrated an OS benefit in pMMR/MSS mCRC, yet this came at the cost of significantly elevated immune-related adverse events compared to supportive care.

Recent real-world evidence reinforces this high-risk profile. In the study by Zhao et al. (74), nearly 40% of patients receiving dual salvage therapy experienced Grade ≥3 treatment-related adverse events (TRAEs). Such toxicity is non-negligible for a palliative population, suggesting that survival advantage is strictly contingent upon the patient’s physiological reserve to withstand the induction phase.

To resolve this impasse, clinical strategy must evolve from simple fitness-based exclusion to multidimensional precision selection. First, pharmacological innovation offers a structural solution: next-generation Fc-engineered CTLA-4 inhibitors, such as Botensilimab, are designed to strip the Fc domain of complement-fixation activity. This effectively decouples efficacy from systemic toxicity (e.g., colitis) while enhancing intratumoral Treg depletion (77). Second, regarding biomarker guidance, identifying the *POLE/POLD1* mutation subset is critical. These ultra mutated tumors exhibit exquisite sensitivity to immunotherapy, potentially permitting de-escalated dosing regimens that mitigate toxicity without compromising survival (78).

Therefore, we advocate for a restrictive and precision-guided protocol. Dual blockade should not be viewed as a standard “next-line” default, but as a specialized intervention reserved for a niche cohort: those who are strictly fit (Eastern Cooperative Oncology Group Performance Status [ECOG PS] 0-1) and, ideally, candidates for novel toxicity-sparing agents or carriers of favorable molecular signatures.

## 4 Precision stratification: the key to efficacy

The clinical utility of late-line ICI activation is currently significantly constrained: in unselected populations, the limited survival benefits often fail to offset the potential toxicity burden. As demonstrated by the LEAP-017 trial, treating unselected pMMR/MSS populations leads to a dilution effect of efficacy, thereby masking the biological activity present in specific responding subgroups (50).

**Figure 2:**
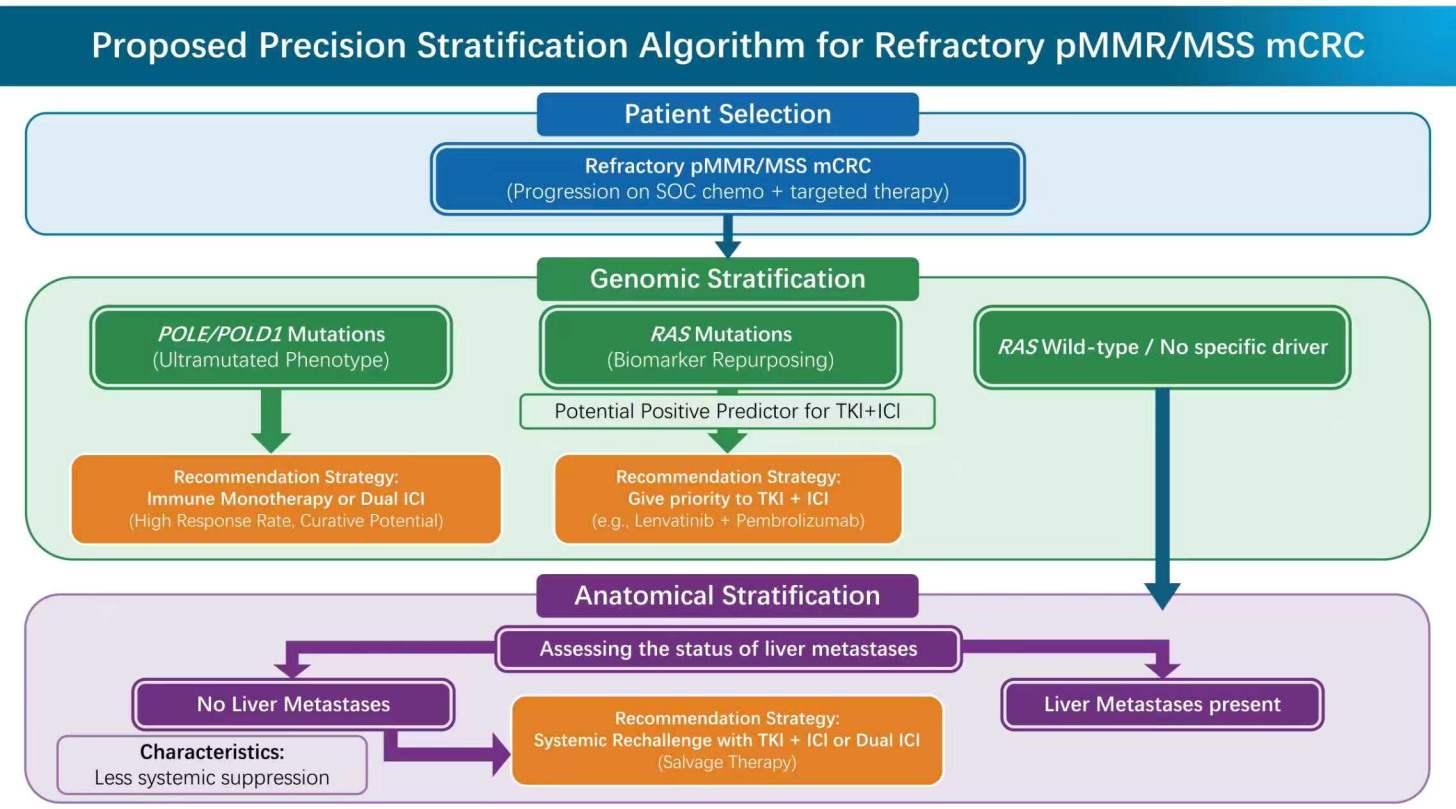
Proposed biomarker-driven precision stratification algorithm for refractory pMMR/MSS mCRC. This flowchart delineates a hierarchical decision-making framework for optimizing immunotherapy rechallenge in the late-line setting. The algorithm prioritizes Genomic Stratification to identify specific hot niches: *POLE*/*POLD1* mutations (ultramutated phenotype) indicate suitability for ICI monotherapy or dual blockade, while *RAS* mutations are repurposed as potential positive predictors for TKI plus ICI combinations. For patients without specific drivers, Anatomical Stratification is employed to address the immune sink effect. Patients without liver metastases are candidates for systemic rechallenge, whereas those with liver metastases require Multimodal Integration incorporating SBRT to mechanically breach the tolerance barrier. The application of intensified regimens, particularly Dual ICI, requires rigorous patient selection based on physiological reserve (ECOG PS 0-1) and autoimmune history to manage toxicity risks.

Consequently, the implementation of biomarker-based stratification is no longer merely an academic exploration but a clinical imperative. Adopting precision medicine strategies is the key pathway to optimizing the “risk-benefit” ratio, ensuring that these intensified strategies are rigorously reserved for subgroups with genuine biological potential for benefit. We propose the construction of a multidimensional stratification framework that integrates genomic drivers and anatomical characteristics to guide clinical guide clinical decision-making (**Figure 2**).

### 4.1 Genomic stratification: identifying the hot niches

Genomic analysis provides a robust foundation for this stratification. Although microsatellite status (MSS *vs*. MSI-H) remains the primary screening criterion, emerging data indicate that within the pMMR/MSS population—previously regarded as homogeneous—specific genomic alterations exist. These alterations can significantly reshape the immunogenic characteristics of the tumor, effectively converting phenotypically cold tumors into functionally hot tumors. Identifying these specific molecular subgroups constitutes the first step in screening for the population with optimal benefit potential.

#### 4.1.1 The *POLE/POLD1* exception: the overlooked ultra mutated niche

Within the vast majority of immune-inert pMMR/MSS populations, there exists a rare but clinically significant subgroup—constituting approximately 0.5%–2% of mCRC patients—harboring mutations in the exonuclease proofreading domains of DNA polymerase ϵ (*POLE*) or δ1 (*POLD1*) (79).

This specific genomic defect leads to the so-called Ultramutated phenotype. Biologically, although these tumors are MSS, their TMB often exhibits an exponential elevation, frequently exceeding that of typical dMMR tumors (80). This genomic instability translates directly into pronounced immunogenicity, manifested by a high load of tumor neoantigen presentation and dense lymphocytic infiltration (81).

For clinicians, this biological feature translates into superior therapeutic sensitivity. Existing evidence indicates that patients with *POLE/POLD1* mutations exhibit excellent responses to ICI therapy (whether as monotherapy or in combination), with survival curves often comparable to those of dMMR patients, achieving long-term or even curative survival (82, 83). Therefore, active screening for *POLE/POLD1* mutations should be regarded as an indispensable step prior to late-line treatment. Missing this biomarker is tantamount to depriving patients of the opportunity for a potentially curative therapy; it is a key beneficiary subgroup that must be identified first in the precision stratification algorithm.

#### 4.1.2 The *RAS* mutation paradox: from negative prognosis to positive prediction

If the *POLE* mutation represents an obvious immune hotspot, the role of *RAS* mutations in combined immunotherapy represents a reshaping of clinical understanding. Historically, *RAS* mutations have been established as negative prognostic factors in mCRC, predicting not only more aggressive biological behavior but also serving as absolute contraindications for anti-EGFR therapy (84, 85).As demonstrated by a recent meta-analysis by Thomsen et al., the negative prognostic impact of *KRAS* mutations extends beyond the metastatic setting, serving as a strong predictor of poor survival outcomes even in early-stage pMMR CRC (86). While the advent of novel agents targeting specific alterations, such as the *KRAS* G12C mutation, has introduced new precision-oncology paradigms, the broader spectrum of *RAS* mutations remains a formidable clinical challenge.

However, in the specific context of combined TKI and ICI application, this gene appears to demonstrate a significant inversion of predictive value. Subgroup analysis of the Phase III LEAP-017 trial provided a critical clue for this paradigm shift: in stark contrast to the negative results in the ITT population, the *RAS*-mutant subgroup achieved a statistically significant and clinically meaningful improvement in PFS (HR 0.57, 95% CI: 0.43 –0.75) when treated with Lenvatinib plus Pembrolizumab (50).

This observation challenges the dogma of *RAS* mutations as purely negative prognostic markers. Biologically, this paradoxical sensitivity likely stems from an intrinsic dependency on the VEGF axis. *RAS*-driven activation of the MAPK pathway constitutively upregulates VEGF-A, fostering a chaotic vascular phenotype that is particularly susceptible to normalization by multi-kinase inhibitors (87). Concomitantly, *RAS* oncogenic signaling reinforces immune exclusion by stabilizing PD-L1 expression (88) and recruiting suppressive myeloid cells (89). Thus, the combination therapy effectively decouples this dual resistance mechanism: the TKI normalizes the *RAS*-induced vascular barrier, paving the way for ICI-mediated T-cell reinvigoration. Consequently, *RAS* genotyping should be reassessed not merely as a marker of aggression, but as a potential predictive biomarker for prioritizing TKI-based immunotherapy.

### 4.2 Anatomical stratification: the liver metastasis factor

Beyond genomic drivers, the anatomical distribution of the tumor—specifically the presence of liver metastases—constitutes another critical dimension of clinical stratification. In the immunotherapy landscape of pMMR/MSS mCRC, the liver is not merely a passive metastatic site; physiologically, it functions as a uniquely tolerogenic organ, exerting a systemic immune sink effect (90).

This biological characteristic stems from the liver’s unique anatomical and physiological requirements: to process antigen-rich blood from the gut without eliciting autoimmune reactions, the liver maintains a highly tolerogenic microenvironment (91–93). However, this mechanism is exploited by tumor metastases, resulting in the capture and clearance of systemic CD8+ T cells by hepatic macrophages (94, 95). The disparity in efficacy presented by clinical data is striking: in pivotal trials such as LEAP-017, the survival benefits of combined immunotherapy were driven almost exclusively by the subgroup without liver metastases, whereas patients with liver metastases often had extremely poor prognoses (50, 96, 97). Therefore, liver metastasis status should not be viewed merely as a marker of poor prognosis, but must be regarded as a primary stratification factor when formulating treatment strategies: for this population, systemic pharmacotherapy alone is often insufficient, necessitating the introduction of multimodal local interventions.

#### 4.2.1 The immune sink effect: a black hole for T cells

The detrimental impact of liver metastases on immunotherapy efficacy stems not merely from increased tumor burden, but from a unique and fatal mechanism of systemic immunomodulation. Pioneering research by Yu et al. (2021) revealed that hepatic metastases remodel the local immune microenvironment, activating liver-specific macrophages (Kupffer cells) (98).

Mechanistically, these activated macrophages act as a filter for systemic CD8+ T cells. They actively capture and clear antigen-specific effector T cells from the systemic circulation. This phenomenon is termed the immune sink effect (98).Importantly, this sink phenomenon appears to be a fundamental property of the hepatic stroma rather than a feature specific to CRC histology. Mechanistic studies across distinct tumor models—ranging from colorectal cancer to melanoma—demonstrate a conserved pathway of macrophage-mediated T-cell elimination (61, 98). This implies that the liver functions as a generic trap for systemic immunity. However, the clinical stakes are highest in CRC given its predominant hepatic tropism. Thus, while our discussion centers on colorectal cancer, the de-sinking strategies proposed herein likely hold broad translational relevance for other liver-tropic malignancies. The consequences are profound: the hepatic lesion effectively becomes a systemic immunosuppressive hub, not only suppressing anti-tumor immunity locally but also, by depleting circulating T cells, causing extrahepatic lesions (such as lung metastases or the primary tumor)—which might otherwise be sensitive to immunotherapy—to lose immune control. This systemic T-cell depletion explains why, in the presence of liver metastases, the systemic efficacy of both PD-1 monotherapy and conventional dual combinations is severely compromised.

It is crucial to distinguish this hepatic sink effect from the immune resistance observed in other metastatic sites, such as the peritoneum. While peritoneal metastases also foster a formidable immunosuppressive niche, their mechanism is predominantly one of local exclusion rather than systemic depletion. Characterized by dense stromal fibrosis and TGF-β-rich ascites, peritoneal tumors create a physical sanctuary that bars T-cell infiltration (99). However, unlike the liver, the peritoneum lacks the hemodynamic architecture—specifically, the filtration of systemic blood circulation—requisite for capturing circulating lymphocytes. Consequently, while peritoneal disease impedes local drug delivery and immune entry, it does not actively sink effector T cells from the systemic pool to the same extent as the liver (98). This mechanistic divergence underscores why liver metastases pose a unique, systemic barrier to immunotherapy that requires distinct remodeling strategies compared to other organ sites.

At the cellular level, this sink effect is orchestrated by a precise elimination machinery rather than a passive void. FasL+ Kupffer cells serve as the primary executioners, physically engaging Fas+ CD8+ T cells within the hepatic sinusoids to trigger immediate apoptosis before they can penetrate the metastatic niche (98). This elimination is reinforced by activated hepatic stellate cells, which secrete SDF-1 (CXCL12) to recruit myeloid-derived suppressor cells via the CXCR4 axis, cementing a profound immunosuppressive matrix (100). Such biological intransigence renders systemic monotherapies insufficient; circulating antibodies cannot easily reverse contact-dependent apoptosis or penetrate this dense stromal barrier. This necessitates local interventions like stereotactic body radiation therapy (SBRT), not merely for tumor debulking, but to physically ablate this immune-hostile architecture and reset the sinusoidal microenvironment.

#### 4.2.2 Clinical decision making: beyond pharmacological limits

Given the aforementioned mechanisms, the presence of liver metastases must alter our treatment algorithm. Data from the LEAP-017 trial provides clear clinical evidence for this stratification: the survival benefit of lenvatinib plus pembrolizumab in this study was confined largely to the subgroup without liver metastases (HR 0.65, 95% CI: 0.42–0.99), while patients with liver metastases failed to derive significant survival prolongation compared to standard of care (50).

This data conveys a clear strategic signal: for pMMR/MSS patients with liver metastases, reliance solely on systemic therapy—whether TKI-mediated microenvironment modulation or dual checkpoint blockade—often faces significant pharmacological limits. As established, the liver functions not merely as a physical barrier but as an active, systemic immune sink that sequesters and depletes circulating T cells. Consequently, this potent tolerogenic environment is capable of negating the immune activation conferred by systemic drugs, rendering purely pharmacological immune rechallenge largely ineffective. Therefore, clinical decision-making for this subgroup must shift toward a multidisciplinary approach. To achieve systemic efficacy, it is a prerequisite to physically disrupt this local tolerance barrier. Intensified local therapies, such as stereotactic body radiation therapy (SBRT), must be introduced to mechanically ablate the suppressive hepatic niche.

To translate these mechanistic insights into actionable clinical strategies, we have formulated a biomarker-driven precision stratification framework, as detailed in **Table 2**. This algorithm systematically categorizes patients into distinct therapeutic tiers by integrating genomic biomarkers (specifically *POLE/POLD1* and *RAS* status) with anatomical characteristics (the presence of liver metastases). By synthesizing the strength of evidence from recent trials, this framework offers graded recommendations ranging from monotherapy to multimodal triple therapy, aiming to guide clinicians in identifying the optimal immune-responsive niches within the heterogeneous pMMR/MSS mCRC population.

**Table 2:** Proposed biomarker-driven precision stratification framework for immunotherapy re-challenge in pMMR/MSS mCRC.

Stratification dimension	Feature/biomarker	Clinical significance	Recommended strategy	Strength of evidence
Genomic	*POLE/POLD1* Mutations	Ultramutated phenotype^a^, extremely high TMB, highly immunogenic (79, 80).	Preferred: ICI monotherapy or combination (PD-1 ± CTLA-4).	High (Similar to dMMR outcomes) (82, 83)
Genomic	*RAS* Mutations	Traditionally poor prognosis, but holds potential predictive value in TKI combinations (84, 85).	Prioritize: TKI + IC.	Moderate (LEAP-017 subgroup analysis) (50)
Anatomical	Absence of Liver Metastases	Less systemic immunosuppression; preserved T-cell reserve.	Viable Options: TKI + ICI or Dual ICI.	Moderate-High (Subgroups from multiple trials) (50, 96)
Anatomical	Presence of Liver Metastases	immune Sink^b^ effect; systemic clearance of effector T cells (90, 98).	Avoid purely pharmacological treatment; Requires combination with SBRT (Triple Therapy^c^).	High (Mechanistic rationale & clinical failure data) (50, 98, 105)

dMMR, mismatch repair-deficient; ICI, immune checkpoint inhibitor; SBRT, stereotactic body radiation therapy; TKI, tyrosine kinase inhibitor; TMB, tumor mutation burden. a Ultramutated phenotype refers to tumors with TMB >100 mut/Mb, typically driven by *POLE* exonuclease domain mutations. b immune Sink describes the systemic depletion of CD8+ T cells sequestered by hepatic macrophages in the presence of liver metastases. c Triple Therapy refers to the multimodal combination of SBRT (induction), TKI (stabilization), and ICI (activation).

However, a note of caution is warranted regarding the clinical translation of the multimodal triple therapy (SBRT + TKI + ICI) recommended in this framework. Currently, evidence supporting this intensive regimen is largely derived from retrospective cohorts, placing it in the early exploratory stage of development. While early signals are encouraging, they do not yet constitute Level I evidence. Notably, recent prospective studies have begun to bridge this gap. The RIFLE trial (NCT04948034), evaluating SBRT combined with fruquintinib and tislelizumab, reported an ORR of 29% and a median PFS of 8.8 months in refractory mCRC (101).Furthermore, a recent prospective observational study published in the *Journal for ImmunoTherapy of Cancer* confirmed that radiotherapy exposure significantly enhances the efficacy of fruquintinib plus PD-1 blockade. Patients in the radiotherapy cohort achieved an ORR of 28.0% compared to only 6.7% in the non-radiotherapy group, with a significantly prolonged PFS (6.2 *vs*. 2.7 months) (102).These findings validate the proof-of-concept, yet large-scale, randomized Phase III trials are imperative to definitively establish the efficacy and safety of adding SBRT to standard TKI+ICI doublets before routine clinical adoption.

### 4.3 Intensification strategy: triple-agent regimens

Beyond standard doublets, triple-agent regimens combining an immune primer with dual checkpoint inhibition (anti-PD-1 plus anti-CTLA-4) are under active investigation to breach the high immune tolerance of pMMR/MSS tumors. This strategy theoretically addresses three rate-limiting steps: TME normalization (via TKI or chemotherapy), T-cell priming (anti-CTLA-4), and effector phase disinhibition (anti-PD-1). Clinical data from two landmark trials illustrate both the potency and the strict selection requirements of this approach.

In the TKI-based setting, Fakih et al. reported that adding Regorafenib to Nivolumab and Ipilimumab yielded an ORR of 36.4% and a median OS exceeding 22 months in refractory pMMR/MSS mCRC (103). Crucially, this benefit was driven entirely by patients without liver metastases; the liver-metastatic cohort derived no benefit (ORR 0%), validating the concept of the liver as a dominant site of immune resistance.

Alternatively, the MAYA trial utilized a molecularly targeted priming strategy. By selecting patients with *MGMT* silencing, investigators used Temozolomide to induce a hypermutated phenotype, thereby sensitizing the tumor to subsequent Nivolumab and Ipilimumab. This regimen achieved an ORR of 45% and a median PFS of 7.0 months (104). These studies collectively indicate that while triple blockade is feasible, its substantial toxicity necessitates precise stratification—limiting its use to fit patients within specific anatomical (non-liver) or molecular (*MGMT*-silenced) niches.

## 5 Future directions: multimodal integration

Having analyzed the therapeutic ceiling of current immune-induction strategies, we now turn to potential future paradigms. It is imperative to strictly delineate the following discussion from established clinical standards. While the preceding sections detailed empirically validated outcomes—namely, the limited efficacy of PD-1 monotherapy in unselected pMMR CRC—the strategies proposed below represent hypothesis-generating frontiers. Concepts such as repurposing *RAS* mutations as predictive biomarkers and integrating SBRT are supported by robust translational rationale and retrospective data (e.g., from the LEAP-017 trial), yet they await validation in prospective, randomized settings. Consequently, these approaches should be viewed as investigational frameworks aimed at transcending the current all-comers impasse through mechanism-driven precision.

### 5.1 SBRT: overcoming physical barriers and inducing immunogenicity

Addressing the systemic treatment resistance caused by the hepatic immune sink effect, the introduction of SBRT represents a highly promising multimodal intervention strategy. In this architecture, the role of SBRT undergoes a paradigm shift: it is no longer merely a palliative measure for local debulking but is redefined as a critical immune sensitizer or *in situ* vaccine (105).

The biological logic of this strategy lies in utilizing high-dose, hypofractionated radiation to induce robust ICD. This controlled cellular disintegration releases large quantities of sequestered tumor neoantigens and DAMPs, thereby inducing a strong pro-inflammatory microenvironment locally (105–108). This physical intervention effectively disrupts the inherent immune tolerance state of the liver, providing the necessary molecular targets for subsequent immune recognition (105).

Its ultimate clinical goal is to induce the abscopal effect, whereby local irradiation triggers a systemic anti-tumor immune response (109, 110). However, given that radiotherapy alone struggles to overcome systemic inhibition, the current clinical frontier favors Triple Therapy, aiming to construct a mechanistic closed loop: utilizing TKIs to normalize vasculature and enhance infiltration; utilizing SBRT to release antigens and initiate recognition; and utilizing ICIs to block negative regulation and sustain activity (111, 112). This physical-chemical synergy constitutes the current rational therapeutic framework for overcoming immune tolerance in liver metastases.

#### 5.1.1 The physical trigger: the *in situ* vaccination effect

The key role of SBRT in immuno-oncology lies in overcoming local immune tolerance. Unlike conventional palliative radiotherapy, SBRT utilizes high-dose, hypofractionated radiation patterns to trigger a specific form of cell death within the tumor—ICD.

This process not only achieves physical debulking but, more importantly, induces a massive antigen release at the molecular level. Irradiated tumor cells release copious amounts of DAMPs as well as previously sequestered tumor neoantigens (105–108). These molecules act as endogenous adjuvants, capable of recruiting DCs to infiltrate the tumor parenchyma and promoting their maturation (113, 114). Essentially, SBRT transforms the tumor into an autologous *in situ* vaccine, initiating the antigen presentation process locally and providing the necessary identification targets for the subsequent immune cascade.

#### 5.1.2 Inducing the abscopal effect: synergy of triple therapy

The core therapeutic goal of SBRT is not limited to control within the radiation field, but rather extends to inducing the abscopal effect—triggering systemic anti-tumor immunity via local treatment to regress non-irradiated distant metastases. However, this phenomenon is infrequently observed with radiotherapy alone, attributed to the containment of newly activated T cells by systemic immunosuppression (115–117).

This constitutes the theoretical basis for Triple Therapy (SBRT + TKI + ICI). Retrospective clinical data indicate that in pMMR/MSS mCRC patients, the combination of radiotherapy with anti-angiogenic and immunotherapeutic agents yields survival benefits significantly superior to dual combinations. Its synergistic mechanism manifests as functional complementarity: SBRT is responsible for antigen release; TKIs are responsible for improving perfusion; and ICIs are responsible for releasing inhibition. This multimodal mechanical-chemical synergy represents the most promising comprehensive strategy currently available to overcome tolerance in liver metastases and achieve systemic control.

### 5.2 Expanding the front: from T-cell exhaustion to host microbiome

Although the SBRT+TKI+ICI triple therapy constructs a comprehensive intervention framework targeting physical barriers and the microenvironment, to achieve more durable efficacy, future strategies must further address the functional exhaustion of effector T cells and the host’s systemic microenvironment.

On one hand, prolonged exposure to immune checkpoint inhibitors inevitably drives T cells into a state of deep exhaustion, characterized by the compensatory upregulation of co-inhibitory receptors, such as T cell immunoreceptor with Ig and ITIM domains (TIGIT), lymphocyte-activation gene 3 (LAG-3), and T-cell immunoglobulin and mucin-domain containing-3 (118, 119). Sole blockade of PD-1 may induce the compensatory upregulation of other inhibitory receptors, thereby limiting efficacy. Consequently, next-generation immunotherapy is shifting toward multidimensional blockade, specifically targeting TIGIT or LAG-3 in combination (120, 121). This strategy aims to reverse T-cell dysfunction, ensuring that they retain effector function after infiltrating the tumor via TKI-remodeled vasculature.

On the other hand, the dimension of treatment is expanding from the local tumor to the systemic host. Emerging evidence indicates that the gut microbiome serves as a key modulator of immunotherapy efficacy (122). Specific commensal bacteria (such as *Akkermansia muciniphila*) not only influence baseline sensitivity to PD-1 blockade but may also modulate treatment-related toxicity (123, 124). However, the microbial landscape governing immune response is a complex ecological network rather than a single-species effect. For instance, *Bifidobacterium* species have been shown to enhance dendritic cell maturation and prime CD8+ T cells via the stimulator of interferon genes (STING) signaling pathway (125).Conversely, the enrichment of *Fusobacterium nucleatum*—a pathogen intrinsically linked to CRC pathogenesis—actively drives resistance. Mechanistically, *F. nucleatum* binds its Fap2 protein to the inhibitory receptor TIGIT on NK and T cells, directly paralyzing their cytotoxic functions (126). Furthermore, small-molecule metabolites like inosine have been identified as potent natural adjuvants that rescue anti-CTLA-4 efficacy via the adenosine A2A receptor (127). Consequently, interventions such as fecal microbiota transplantation (FMT) or probiotic modulation are evolving from mere supportive care into active immune adjuvants or sensitizers, aiming to assist anti-tumor immunity by reshaping the host metabolic environment (124, 128).

Translating these mechanistic concepts into clinical practice remains the ultimate goal. While earlier studies in melanoma provided the first proof-of-principle that FMT could reverse anti-PD-1 resistance by reprogramming the tumor microenvironment (129),evidence in gastrointestinal malignancies has been slower to emerge. However, recent data have begun to validate this approach in refractory solid tumors. A prospective trial published in *Cell Host & Microbe* (2024) demonstrated that FMT combined with anti-PD-1 therapy induced objective responses and achieved a disease control rate of 46.2% in a heavily pretreated cohort, which included patients with colorectal cancer (130). For pMMR/MSS mCRC, a phenotype characterized by a paucity of spontaneous T-cell infiltration, these findings suggest that ecosystem transfer is not merely theoretical but a viable strategy to ignite an anti-tumor immune response.

### 5.3 Limitations and translational challenges

The translation of this precision stratification framework from theory to routine practice confronts several immediate realities. Logistically, the reliance on comprehensive genomic profiling restricts accessibility; testing for rare *POLE/POLD1* mutations or accurately quantifying TMB requires standardized assays (78) that are often unavailable in community oncology settings. Furthermore, the proposed multimodal integration—specifically the coordination of SBRT with systemic therapy—demands a level of multidisciplinary synchrony that is difficult to replicate outside of specialized academic centers (131).

Economically, the burden of these intensified regimens is profound. Combining brand-name TKIs with dual checkpoint inhibitors introduces significant financial toxicity (132), potentially limiting patient access in resource-constrained healthcare systems. Regulatorily, it must be acknowledged that the repurposing of *RAS* mutations as positive predictors currently rests on retrospective subgroup analyses. Until these signals are validated in prospective trials, such biomarker-driven strategies remain off-label interventions (133). Therefore, clinical application requires rigorous shared decision-making, carefully balancing the scientific promise of immune activation against the certainty of increased cost and toxicity.

## 6 Conclusion: from empiricism to stratified precision

Breaking the immune tolerance of pMMR/MSS mCRC requires a departure from the one-size-fits-all empiricism that has defined the last decade. The analysis presented here suggests that clinical resistance is not monolithic but stems from identifiable mechanistic barriers—specifically, vascular exclusion and priming failure.

The stratification framework proposed in this review, which integrates genomic repurposing (utilizing *RAS* and *POLE* status) with anatomical filtering (liver metastasis management), offers a biologically grounded roadmap to dismantle these barriers. However, it is critical to emphasize that this framework currently represents an investigational paradigm. While supported by translational rationale and retrospective signals, strategies such as *RAS*-guided TKI selection or multimodal SBRT integration await validation in prospective, randomized trials and should not yet supersede established standard-of-care guidelines outside of clinical research.

For the practicing clinician, the immediate realistic implication is the necessity of patient selection. Moving forward, the decision to deploy salvage immunotherapy must shift from random attempts to rational targeting—prioritizing patients with favorable genomic profiles and physiological reserve (ECOG PS 0-1) while sparing others from the toxicity of ineffective interventions. Ultimately, the goal is to convert the serendipitous responders of the past into the predictable beneficiaries of the future.

## Glossary

ADCC: Antibody-Dependent Cell-Mediated Cytotoxicity
Akt: Protein Kinase B
APC: Antigen-Presenting Cell
CI: Confidence Interval
CSF1R: Colony Stimulating Factor 1 Receptor
CTL: Cytotoxic T Lymphocyte
DAMP: Damage-Associated Molecular Patterns
DC: Dendritic Cells
dMMR: Mismatch Repair-Deficient
ECOG PS: Eastern Cooperative Oncology Group Performance Status
EGFR: Epidermal Growth Factor Receptor
FGFR: Fibroblast Growth Factor Receptor
FMT: Fecal Microbiota Transplantation
HR: Hazard Ratio
ICD: Immunogenic Cell Death
ICI: Immune Checkpoint Inhibitor
irAE: Immune-Related Adverse Event
ITT: Intention-to-Treat
LAG-3: Lymphocyte-Activation Gene 3
MAPK: Mitogen-Activated Protein Kinase
mCRC: Metastatic Colorectal Cancer
MDT: Multidisciplinary Treatment
MGMT: O(6)-Methylguanine-DNA Methyltransferase
MSI-H: Microsatellite Instability-High
MSS: Microsatellite Stable
mTOR: Mammalian Target of Rapamycin
NK: Natural Killer
ORR: Objective Response Rate
OS: Overall Survival
PD-1: Programmed Cell Death Protein 1
PD-L1: Programmed Death-Ligand 1
PFS: Progression-Free Survival
PI3K: Phosphoinositide 3-Kinase
pMMR: Mismatch Repair-Proficient
POLD1: DNA Polymerase Delta 1
POLE: DNA Polymerase Epsilon
RCT: Randomized Controlled Trial
SBRT: Stereotactic Body Radiation Therapy
SDF-1: Stromal Cell-Derived Factor 1
SOC: Standard of Care
STING: Stimulator of Interferon Genes
TAS-102: Trifluridine/Tipiracil
TGF-β: Transforming Growth Factor Beta
TIGIT: T Cell Immunoreceptor with Ig and ITIM Domains
TIM-3: T-Cell Immunoglobulin and Mucin-Domain Containing-3
TMB: Tumor Mutation Burden
TRAE: Treatment-Related Adverse Event
Treg: Regulatory T Cell
VEGF: Vascular Endothelial Growth Factor
VEGFR: Vascular Endothelial Growth Factor Receptor
